# Awareness Level of Herpes Zoster Infection Among the Residents of Jeddah, Saudi Arabia

**DOI:** 10.7759/cureus.101941

**Published:** 2026-01-20

**Authors:** Reem A Algarni, Lama A Rammal, Amira M Marei, Anfal Saber, Raghad Alhajrasi, Laura Althuniyyan, Mawdah Hindi, Shahad Alzahrani

**Affiliations:** 1 Department of Family Medicine, Primary Health Care, Western Region, King Abdulaziz Medical City, Jeddah, SAU; 2 College of Medicine, King Saud Bin Abdulaziz University for Health Sciences, Jeddah, SAU; 3 Department of Obstetrics and Gynecology, King Abdulaziz Medical City, Riyadh, SAU; 4 Department of Family Medicine, King Faisal Specialist Hospital and Research Center, Jeddah, SAU; 5 Department of Family Medicine, Directorate of Health Affairs, Jeddah, SAU

**Keywords:** awareness, knowledge, shingles, vaccination, herpes zoster

## Abstract

Introduction: Herpes zoster (HZ), also known as shingles, is a viral infection caused by the reactivation of the varicella-zoster virus. Awareness and knowledge of HZ and its associated complications vary across countries. Limited data are available on the awareness and knowledge of HZ among the general population in Saudi Arabia. Therefore, this study aims to measure awareness of HZ infection and its vaccine among the citizens of Jeddah, Saudi Arabia.

Methods: An online questionnaire was distributed to the citizens of Jeddah, Saudi Arabia, to assess their awareness and knowledge of HZ. The questionnaire included demographic characteristics, awareness of HZ and its vaccine, and knowledge about both HZ and the HZ vaccine. Descriptive statistics and logistic regression analysis were used to analyze the data.

Results: A total of 557 valid responses were included in the analysis. The majority were female participants (79%) and Saudi nationals (94.8%). The awareness level of HZ was high, with 85.1% of participants reporting having heard of it. However, only 34.2% were aware of the HZ vaccine. The knowledge level regarding HZ and its vaccine varied, with some misconceptions observed. Factors associated with HZ vaccination included age, family history of HZ, and awareness of HZ. Participants with a family history of HZ had higher odds of vaccination (OR: 4.2, p = 0.002).

Conclusion: This study revealed moderate awareness and knowledge of HZ and its vaccine among citizens of Jeddah, Saudi Arabia. Improving knowledge about HZ and its vaccine can contribute to the prevention and management of this viral infection.

## Introduction

Herpes zoster (HZ), or shingles, is a viral infection caused by reactivation of the varicella-zoster virus (VZV). As there is a decline in cell-mediated immunity to VZV with age, it reactivates the virus. This leads to HZ, which is characterized by a maculopapular or vesicular rash and dermatomal distribution pain [1,2]. Patients who are above the age of 50, immunocompromised, or receiving immunosuppressive therapy are more prone to HZ [3]. Abnormal skin sensations and pain of varying severity are the most common symptoms of HZ. This is followed by zoster rash, characterized by vesicles that are unilaterally distributed within a dermatome and do not cross the midline [2-4]. Complications of HZ include secondary bacterial infection, postherpetic neuralgia (PHN), scarring, nerve palsy, and encephalitis in the case of disseminated zoster [2].

Worldwide, awareness of HZ among individuals screened varies markedly according to various countries, ranging from 97% to 100% in New Zealand, Brazil, and Malaysia to less than 20% in Turkey, India, and Chile [5]. Furthermore, several studies have reported in the Gulf Cooperation Council with a prevalence ranging from 15% to 92.2% in Saudi Arabia, the United Arab Emirates, and Qatar. However, no data were available for Bahrain, Kuwait, or Oman [6]. Locally, from January 2014 to August 2021, in the Family Medicine Department at King Faisal Specialist Hospital and Research Center in Riyadh, 330 cases of HZ were identified [7]. In the Eastern Region, 141 of 22,749 cases in the dermatology clinics were found to have HZ, representing 0.62% [8]. Specifically, in Al Khobar, during a five-year study period (January 2010-December 2014) at the dermatology clinic of King Fahad Hospital of the University, HZ accounted for 7.7% of cases, with no specific age distribution [9]. The differences in these percentages may be due to the high level of involvement of primary healthcare (PHC) centers in managing simple, uncomplicated viral illnesses.

Vaccination against HZ is an effective way to prevent HZ and its associated complications [10]. It can reduce the incidence and severity of both HZ and PHN and the disease burden [11]. The United States Food and Drug Administration approved two vaccines, SHINGRIX® (GlaxoSmithKline plc, London, UK) and Zostavax® (Merck & Co., Inc., Rahway, NJ). The vaccine's protection period may last for at least 7-10 years [11]. The Ministry of Health (MOH) in Saudi Arabia announced the availability of the HZ vaccine at all PHC centers, administered in two doses two to six months apart [8]. The MOH recommends that individuals aged 50 years and older, or those aged 18 years and older if diagnosed with autoimmune diseases or taking immunosuppressant medication, receive the vaccine [12].

To our knowledge, no previous studies have assessed the public awareness and knowledge of HZ infection and its vaccine in Jeddah, Saudi Arabia. Therefore, this study aimed to assess the level of awareness and knowledge regarding HZ infection and its vaccine among the citizens of Jeddah, Saudi Arabia, and to identify factors associated with HZ vaccination uptake and intention to vaccinate.

## Materials and methods

Study design and setting

An observational, quantitative, cross-sectional study design was employed. The study was conducted in Jeddah, Saudi Arabia, in 2023 using an online questionnaire to assess public awareness regarding HZ infection.

Inclusion and exclusion criteria

The study included both male and female citizens of the Jeddah region, aged 12 years or older and literate. Individuals who were not citizens of Jeddah were excluded.

Sample size and sampling technique

The study sample size was calculated by using the "Raosoft" software (Raosoft, Inc., Seattle, WA). A minimum targeted sample size was 385, which was calculated based on a 5% margin of error, 95% confidence level, and based on the estimated population of Jeddah. A convenience sampling technique was used. All eligible individuals from the general population of Jeddah who met the inclusion criteria and had access to the online questionnaire during the study period were invited to participate. Although the study targeted the general population of Jeddah, participation was voluntary and limited to respondents who chose to complete the online survey.

Data collection and management plan

Data were collected through an online, self-administered questionnaire targeting the general population in Jeddah. The questionnaire was adapted from instruments previously used in peer-reviewed studies [13], with additional items added to improve relevance to the study objectives and local context. Formal psychometric validation of the adapted questionnaire was not performed prior to data collection. A complete copy of the questionnaire is provided in the Appendix.

Following Institutional Review Board (IRB) approval from King Abdullah International Medical Research Center (KAIMRC), responses were collected and stored in Microsoft Excel 2010 (Microsoft Corporation, Redmond, WA) and analyzed using the Statistical Package for the Social Sciences (SPSS) version 24.0 (IBM Corp., Armonk, NY). Qualitative variables were summarized using frequencies and percentages, and bar charts, whereas quantitative variables were presented as mean ± standard deviation. The chi-square test was used to compare qualitative variables, and univariate and multivariable logistic regression were used to estimate risk factors. The level of significance was determined at p <0.05.

Ethical considerations

Ethical approval was obtained from the IRB of KAIMRC. Participation was voluntary, and informed consent was obtained via an online form that outlined the study's purposes and ensured confidentiality. No personal identifiers were collected. Data were securely stored on a password-protected workplace computer, accessible only to the principal investigator and co-investigators. All data were handled and presented ethically, with no fabrication or falsification.

Statistical analysis

Statistical analysis was performed using SPSS version 29.0 (IBM Corp., Armonk, NY) to identify predictors of HZ vaccination and intention to vaccinate. Descriptive statistics summarized demographic characteristics and knowledge responses as frequencies and percentages. Age was dichotomized (<50 vs. ≥50) based on national vaccination guidelines.

Bivariate logistic regression was used to assess associations between key variables (e.g., age, gender, education, city, history of HZ, and awareness) and outcomes. Variables with p < 0.10 were included in a multivariate logistic regression model, adjusted for age, gender, education, and awareness. Results were reported as odds ratios (ORs) with 95% confidence intervals. For the intention-to-vaccinate analysis, individuals already vaccinated were excluded, focusing the model on unvaccinated participants to identify influencing factors.

## Results

A total of 557 valid responses were included in the statistical analysis. Table 1 presents the sociodemographic and herpes-related history of the participants in the study. The age distribution of the participants shows that the majority were in the age range of 20-39 (44.4%) and 41-59 (41.7%) years. The gender distribution indicates that 79% were female participants. Regarding nationality, 94.8% of the participants were Saudi. In terms of educational level, the majority had a university degree (72.2%). The prevalence of comorbidities among the participants was relatively high, with hypertension (12.9%), diabetes mellitus (14.7%), and gastrointestinal diseases (10.6%) being the most common.

**Table 1:** Sociodemographic and herpes-related history of the participants HZ: herpes zoster

Variables	Groups	n (%)
Age	<20	20 (4.10%)
	20-39	214 (44.40%)
	41-59	201 (41.70%)
	≥60	47 (9.80%)
Gender	Male	101 (21.00%)
	Female	381 (79.00%)
Nationality	Saudi	457 (94.80%)
	Non-Saudi	25 (5.20%)
Educational level	Elementary or less	2 (0.40%)
	High school or less	93 (19.30%)
	University	348 (72.20%)
	Higher education	39 (8.10%)
Comorbidities	Hypertension	62 (12.90%)
	Diabetes mellitus	71 (14.70%)
	Cancer	14 (2.90%)
	Gastrointestinal diseases	51 (10.60%)
	Kidney diseases	9 (1.90%)
	Cardiovascular diseases	7 (1.50%)
	Dyslipidemia	69 (12.40%)
Past history of chickenpox	Yes	280 (58.10%)
	No	168 (34.90%)
	Not sure	34 (7.10%)
Past history of HZ	Yes	28 (5.80%)
	No	443 (91.90%)
	Not sure	11 (2.30%)
Family history of HZ	Yes	154 (32.00%)
	No	328 (68.00%)
Heard of HZ virus	Yes	410 (85.10%)
	No	72 (14.90%)
Heard of the HZ vaccine	Yes	165 (34.20%)
	No	317 (65.80%)
Flu vaccination last year	Yes	275 (57.10%)
	No	207 (42.90%)
City of residence	Jeddah	418 (86.70%)
	Others	64 (13.30%)
Know a person diagnosed with HZ	Yes	280 (58.10%)
	No	168 (34.90%)
Do you know that the HZ vaccine is recommended for people aged 50 years or older?	Yes	266 (47.80%)
	No	291 (52.20%)

In regard to the participants' herpes-related history, 280 (58.1%) participants reported a past history of chickenpox, while only 28 (5.8%) participants reported a past history of HZ. A significant number of participants (32%) had a family history of HZ. When asked about their awareness of the HZ virus, 410 (85.1%) participants reported having heard of it, while 165 (34.2%) participants reported being aware of the HZ vaccine. In terms of knowing a person diagnosed with HZ, 280 (58.1%) participants responded positively, while 168 (34.9%) responded negatively. Regarding awareness that the HZ vaccine is recommended for people aged 50 years or older, 266 (47.8%) participants were aware, whereas 291 (52.2%) were not.

Table 2 presents the participants' knowledge regarding the HZ virus. Around 369 (76.6%) participants were unsure if an individual who had chickenpox would be at risk of HZ. Regarding the risk of HZ in immunocompromised individuals, 264 (54.8%) participants were unsure, while 212 (44%) believed that immunocompromised individuals were at a higher risk. When asked if young people could have HZ, 277 (57.5%) participants were unsure, while 88 (18.3%) believed that young people would not have HZ. In terms of acquiring HZ from contact with HZ patients, 243 (50.4%) participants were unsure, while 96 (19.9%) believed that individuals who had contact with HZ patients would acquire HZ. Furthermore, 255 (52.9%) participants were unsure whether any drugs were available for treating HZ.

**Table 2:** Participants’ knowledge regarding HZ virus HZ: herpes zoster

Statement	True, n (%)	False, n (%)	I don’t know, n (%)
If an individual had chickenpox virus, he/she will be at risk of HZ	87 (18%)	26 (5.4%)	369 (76.6%)
Immunocompromised individuals are at a higher risk of HZ	212 (44%)	6 (1.2%)	264 (54.8%)
Young people will not have HZ	88 (18.3%)	117 (24.3%)	277 (57.5%)
Individuals who have contact with HZ patients will acquire HZ	96 (19.9%)	143 (29.7%)	243 (50.4%)
There are no drugs available for treating HZ	58 (12%)	169 (35.1%)	255 (52.9%)

**Figure 1:**
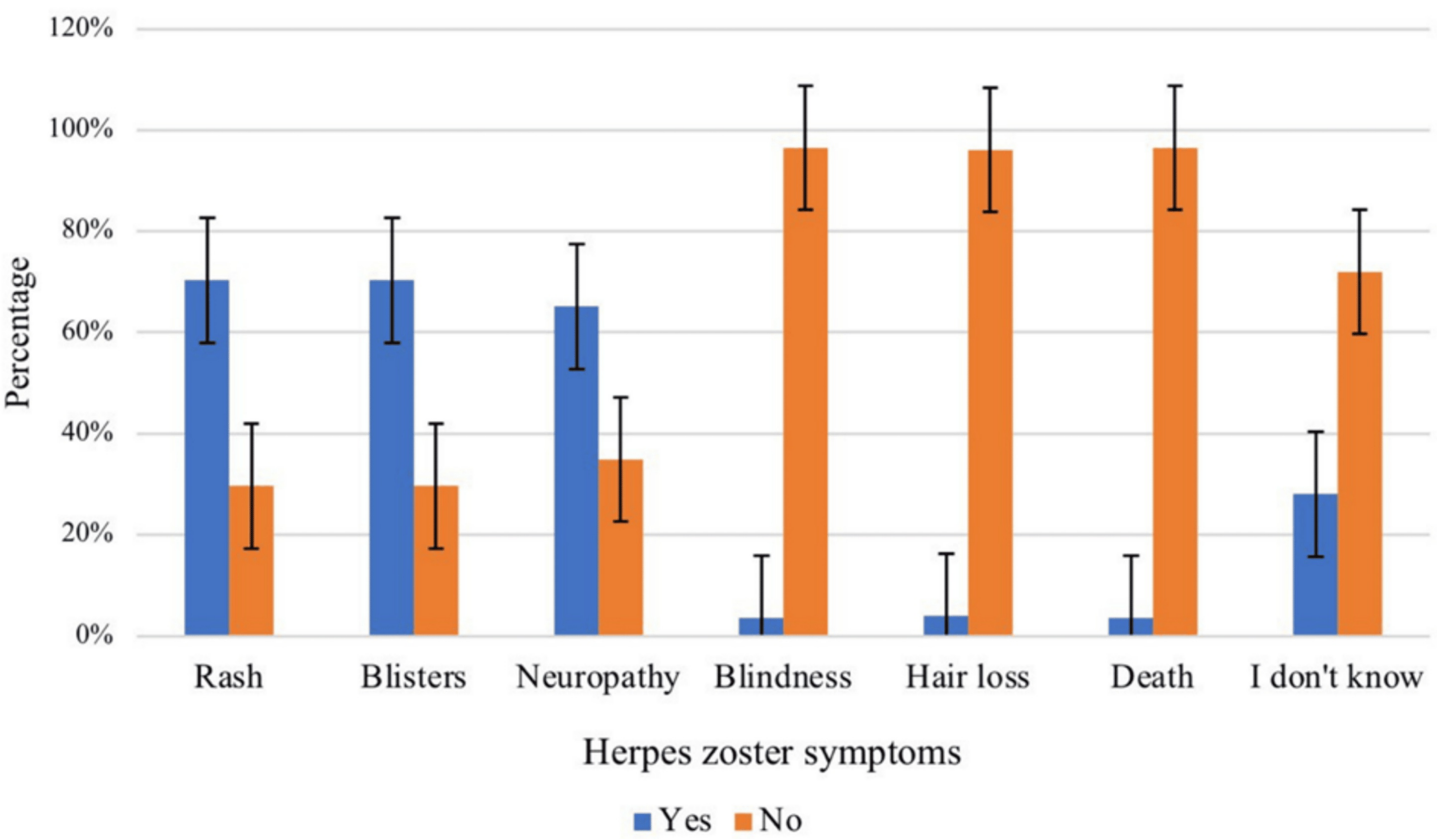
Participants’ knowledge regarding herpes zoster symptoms

Figure 1 presents the participants' knowledge regarding HZ symptoms. The majority of participants were aware of the symptoms, with 70.3% correctly identifying rash and blisters as HZ symptoms. Additionally, 65.1% identified neuropathy as a symptom. However, there were misconceptions among some participants, with a small percentage incorrectly associating HZ with blindness, hair loss, and death.

Table 3 shows the responses to knowledge questions. According to the data, 288 (47.3%) participants were aware that the HZ vaccine can reduce disease incidence by more than 50%. Additionally, 91 (18.9%) participants believed that the HZ vaccine can treat active HZ. Regarding the approved age group for vaccination against HZ, 233 (48.3%) participants correctly identified that it is recommended for individuals aged 50 years or older. It is worth noting that 64 (13.3%) participants reported being recommended to get the HZ vaccine. However, the majority of participants (94.4%) have not received the HZ vaccine.

**Table 3:** Responses to HZ vaccine knowledge questions HZ: herpes zoster

Statement	Response	n (%)
The HZ vaccine can reduce the incidence of disease by >50%	True	228 (47.30%)
	False	2 (0.40%)
	I don’t know	252 (52.30%)
The HZ vaccine can treat active HZ infection	True	91 (18.90%)
	False	70 (14.50%)
	I don’t know	321 (66.60%)
Which age group is approved for vaccination against HZ?	No specific age	40 (8.30%)
	13-26 years	3 (0.60%)
	18 years and older	23 (4.80%)
	50 years and older	233 (48.30%)
	I don’t know	183 (38.00%)
Did you receive the HZ vaccine?	Yes	27 (5.60%)
	No	455 (94.40%)
Do you have the intention to get the HZ vaccine?	Yes	283 (62.20%)
	No	172 (37.80%)
Have you been recommended by a healthcare practitioner to get the HZ vaccine?	Yes	64 (13.30%)
	No	418 (86.70%)

As shown in Table 4, bivariate analysis revealed several significant factors associated with HZ vaccination status. Hypertension was the only comorbidity significantly associated with vaccination (14.5% of hypertensive participants were vaccinated, p = 0.004), while other comorbidities showed higher vaccination rates without reaching statistical significance. Awareness and behavioral factors were strongly associated with vaccination status, including knowledge of the HZ vaccine (11% vs. 0.4%, p < 0.001), awareness of age-related vaccination recommendations (9.9% vs. 1.6%, p < 0.001), and receiving flu vaccination in the previous year (10.9% vs. 2.8%, p < 0.001). Personal connections to HZ also showed significant associations, with higher vaccination rates among those with a family history of HZ (11.7% vs. 2.7%, p < 0.001) and those who knew someone diagnosed with HZ (7.6% vs. 2.9%, p = 0.025). The age distribution showed an increasing trend in vaccination rates with age (21.3% in individuals aged ≥60 years), although this did not reach statistical significance (p = 0.104).

**Table 4:** Bivariate analysis of potential predictors to HZ vaccination ^*^p value was calculated using Fisher’s exact test GI: gastrointestinal; CVD: cardiovascular disease; HZ: herpes zoster

Variables	Groups	Total	Vaccination status		p value
			Vaccinated	Not vaccinated	
Age	<20	20 (4.1%)	0 (0%)	20 (100%)	0.104
	20-39	214 (44.4%)	5 (2.3%)	209 (97.7%)	
	41-59	201 (41.7%)	12 (6%)	189 (94%)	
	≥60	47 (9.8%)	10 (21.3%)	37 (78.7%)	
Gender	Male	101 (21%)	9 (8.9%)	92 (91.1%)	0.140^*^
	Female	381 (79%)	18 (4.7%)	363 (95.3%)	
Nationality	Saudi	457 (94.8%)	27 (5.9%)	430 (94.1%)	0.386^*^
	Non-Saudi	25 (5.2%)	0 (0%)	25 (100%)	
Educational level	Elementary or less	2 (0.4%)	0 (0%)	2 (100%)	0.354^*^
	High school or less	93 (19.3%)	3 (3.2%)	90 (96.8%)	
	University	348 (72.2%)	20 (5.7%)	328 (94.3%)	
	Higher education	39 (8.1%)	4 (10.3%)	35 (89.7%)	
Comorbidities	Hypertension	62 (12.9%)	9 (14.5%)	53 (85.5%)	0.004^*^
	Diabetes Mellitus	71 (14.7%)	7 (9.9%)	64 (90.1%)	0.097^*^
	Cancer	14 (2.9%)	2 (14.3%)	12 (85.7%)	0.183^*^
	GI diseases	51 (10.6%)	6 (11.8%)	45 (88.2%)	0.054^*^
	Kidney diseases	9 (1.9%)	2 (22.2%)	7 (77.8%)	0.085^*^
	CVD	7 (1.5%)	1 (14.3%)	6 (85.7%)	0.334^*^
	Dyslipidemia	69 (12.4%)	5 (7.8%)	59 (92.2%)	0.384^*^
Past history of chickenpox	Yes	280 (58.1%)	13 (4.6%)	267 (95.4%)	0.487
	No	168 (34.9%)	11 (6.5%)	157 (93.5%)	
	Not sure	34 (7.1%)	3 (8.8%)	31 (91.2%)	
Past history of HZ	Yes	28 (5.8%)	1 (3.6%)	27 (96.4%)	1.000
	No	443 (91.9%)	26 (5.9%)	417 (94.1%)	
	Not sure	11 (2.3%)	0 (0%)	11 (100%)	
Family history of HZ	Yes	154 (32%)	18 (11.7%)	136 (88.3%)	<0.001
	No	328 (68%)	9 (2.7%)	319 (97.3%)	
Heard of the HZ virus	Yes	410 (85.1%)	25 (6.1%)	385 (93.9%)	0.403^*^
	No	72 (14.9%)	2 (2.8%)	70 (97.2%)	
Heard of HZ vaccine	Yes	236 (49%)	26 (11%)	210 (89%)	<0.001
	No	246 (51%)	1 (0.4%)	245 (99.6%)	
Flu vaccination last year	Yes	165 (34.2%)	18 (10.9%)	147 (89.1%)	<0.001
	No	317 (65.8%)	9 (2.8%)	308 (97.2%)	
Know a person diagnosed with HZ	Yes	275 (57.1%)	21 (7.6%)	254 (92.4%)	0.025
	No	207 (42.9%)	6 (2.9%)	201 (97.1%)	
Do you know that the HZ vaccine is recommended for people aged 50 years or older?	Yes	232 (48.1%)	23 (9.9%)	209 (90.1%)	<0.001
	No	250 (51.9%)	4 (1.6%)	246 (98.4%)	

Table 5 provides valuable insights into the factors associated with HZ vaccination. The results of the multivariate logistic regression analysis reveal some significant ORs that can help us understand which groups tend to be vaccinated against HZ. Participants aged <50 years had significantly lower odds of vaccination (OR = 0.18, p < 0.001). Male participants showed higher odds of being vaccinated than female participants (OR = 1.97, p = 0.110). When considering the participants' educational level, those with a high school education or less and university educational levels had significantly lower odds of being vaccinated than those with higher education (OR = 0.29, p = 0.112; OR = 0.53, p = 0.275), respectively. Interestingly, a family history of HZ was significantly associated with a higher odds of vaccination (OR = 4.7, p < 0.001). This suggests that individuals with a family history of HZ are more likely to get the vaccination. Awareness and past history of HZ did not show a statistical association with vaccination. Significant predictors were used in a new model to define significant predictors after adjustment. Age, family history of HZ, and awareness of HZ remained significant predictors after adjusting for age, gender, educational level, and awareness of HZ.

**Table 5:** Factors associated with HZ vaccination ^*^Multivariate regression was done while controlling for age, educational level, family history of HZ, and awareness of HZ OR: odds ratio; CI: confidence interval; HZ: herpes zoster

Variables		Univariate logistic regression		Multivariate logistic regression^*^	
		OR (95% CI)	p value	OR (95% CI)	p value
Age	<50	0.18 (0.08-0.41)	<0.001	0.21 (0.09-0.48)	<0.001
	≥50	Reference	-	-	-
Gender	Male	1.97 (0.86-4.5)	0.110	-	-
	Female	Reference	-	-	-
Educational level	High school or less	0.29 (0.06-1.3)	0.112	-	-
	University	0.53 (0.17-1.7)	0.275	-	-
	Higher education	Reference	-	-	-
Family history of HZ	Yes	4.69 (2.1-10.7)	<0.001	4.2 (1.7-10.3)	0.002
	No	Reference	-	-	-
Past history of HZ	Yes	0.61 (0.08-4.7)	0.634	-	-
	No	Reference	-	-	-
Awareness of HZ	Yes	30.3 (4.1-225.4)	0.004	0.7 (0.14-3.512)	0.665
	No	Reference	-	-	-

Table 6 presents the factors associated with the intention to get HZ vaccination among those who were not vaccinated. The results of the univariate logistic regression analysis show that educational level, family history of HZ, and awareness of HZ were significantly associated with the intention to get vaccinated. Participants with a high school education or less had lower odds of intending to be vaccinated than those with higher education (OR = 0.41, p = 0.044). Additionally, participants with a family history of HZ had higher odds of intending to get vaccinated (OR = 1.7, p = 0.016). It is worth noting that awareness of HZ was significantly associated with the intention to be vaccinated, with participants who were aware of HZ having higher odds of intending to be vaccinated (OR = 2.4, p < 0.001). After adjusting for educational level, family history of HZ, and awareness of HZ, the multivariate logistic regression analysis showed that the significant predictors of intention to get vaccinated were educational level and awareness of HZ. Participants with a high school education or less had lower odds of intending to be vaccinated than those with higher education (OR = 0.43, p = 0.058). Similarly, participants who were aware of HZ had higher odds of intending to be vaccinated (OR = 2.1, p = 0.008).

**Table 6:** Factors associated with intention to get HZ vaccination among those who were not vaccinated ^*^Multivariate regression was done while controlling for educational level, family history of HZ, and awareness of HZ OR: odds ratio; CI: confidence interval; HZ: herpes zoster

Variables		Univariate logistic regression		Multivariate logistic regression^*^	
		OR (95% CI)	p value	OR (95% CI)	p value
Age	<50	0.94 (0.61-1.4)	0.765	-	-
	≥50	Reference	-	-	-
Gender	Male	1.2 (0.73-1.9)	0.504	-	-
	Female	Reference	-	-	-
Educational level	High school or less	0.41 (0.17-0.98)	0.044	0.43 (0.18-1.03)	0.058
	University	0.59 (0.27-1.3)	0.194	0.56 (0.25-1.2)	0.153
	Higher education	Reference	-	-	-
Family history of HZ	Yes	1.7 (1.1-2.6)	0.016	1.4 (0.92-2.3)	0.115
	No	Reference	-	-	-
Past history of HZ	Yes	0.88 (0.39-1.9)	0.746	-	-
	No	Reference	-	-	-
Awareness of HZ	Yes	2.4 (1.4-3.9)	<0.001	2.1 (1.2-3.6)	0.008
	No	Reference	-	-	-

## Discussion

This study aimed to assess the level of awareness of HZ infection among residents of Jeddah, Saudi Arabia. The study also assessed age, gender, educational level, and family history. The results of the present study indicate an association between awareness of HZ and greater willingness to receive vaccination (p = 0.031). This finding is consistent with a recent study conducted by AlMuammar et al., which reported that 57.2% of the participants were aware of the HZ vaccination. Among those participants, 53.2% expressed willingness to be vaccinated [14].

A recent study conducted in the western region of Saudi Arabia to assess the knowledge, attitudes, and practices regarding HZ vaccination found that the general Saudi population had good awareness and understanding of HZ vaccination. Awareness was reported by 55.8% of participants, but 94.6% had not received the HZ vaccination. The percentage willing to receive vaccination was 77.4%, whereas 28.1% were unwilling [15]. The results of this study were in line with those of the present study. Another cross-sectional study, performed in Makkah to assess the knowledge of physicians, elaborated that qualification levels and Saudi Commission for Health Specialist classification were significantly associated with knowledge scores of shingles (p = 0.002 and p = 0.003), respectively [16].

In the present study, higher educational levels were also a significant predictor of intention to get vaccinated. Participants with a high school education or less had lower odds of intending to be vaccinated than those with higher education (p = 0.078). However, the results of the study by AlMuammar et al. contradicted this finding. They reported that people with a primary education were 16.1 times more likely to accept vaccination than those with higher educational levels (p = 0.01) [14]. A similar study reported that higher educational levels are associated with increased vaccine hesitancy. This hesitancy can be defined as a refusal or delay of vaccination despite the availability of vaccination services [17]. However, some previous studies have found that people with lower educational levels are at a disadvantage in the form of health literacy. This disadvantage might impact their ability to understand and act on health information, such as vaccination [18,19]. This might be due to less access to healthcare services and less motivation to take advantage of preventive healthcare measures.

In their study, Al-Regaiey et al. reported no association between educational attainment and vaccine hesitancy [20]. However, the results of the studies conducted in Saudi Arabia and Hong Kong found that higher educational levels are associated with greater HZ acceptance [13,21]. This association underscores the importance of education and the provision of primary sources of information to the public in minimizing the spread of misinformation and distorted facts. The results of the present study also indicated a significantly lower intention of vaccination among participants aged less than 50 years (p < 0.001). Similarly, male participants had a higher intention to receive HZ vaccination than female participants (p = 0.087). These findings are in line with the findings of the study of AlMuammar et al., which showed that people aged 56 and above are 11.8 times more willing to accept vaccination. Similarly, they also found that men were 1.9 times more willing to be vaccinated than women (p = 0.01) [14]. Given the cross-sectional design of the study, causal relationships cannot be inferred.

This study has several limitations that should be considered when interpreting the findings. First, the study relied on self-reported data, which may introduce personal bias. Second, it did not explore the effects of social and cultural factors on vaccine acceptance. Third, the demographic distribution was not uniform: the majority of participants were female, and approximately 80% had higher education, which may have influenced the findings, as individuals with higher education generally have better access to information and knowledge. Additionally, the study was conducted in a single region, limiting the generalizability of the results. The use of a convenience sampling technique may further limit representativeness, as participation was voluntary and limited to individuals who chose to complete the online questionnaire; future studies may consider probability-based sampling methods, such as stratified random sampling, to enhance generalizability. Finally, the questionnaire used in this study was adapted from instruments previously employed in peer-reviewed research [13] with additional items included for relevance; formal psychometric validation of the adapted questionnaire was not performed prior to its use, which should be considered when interpreting the findings.

## Conclusions

HZ is a viral infection that occurs due to the reactivation of the VZV. The clinical symptoms range from painful rashes to serious complications like PHN, vision, or hearing loss, and, in rare instances, neurological damage. From a policy perspective, it is recommended that national immunization guidelines include the HZ vaccine as a standard for adults aged 50 and older, as well as for individuals who are immunocompromised, regardless of their age. The vaccine consists of two doses of recombinant zoster vaccine administered two to six months apart. Education and awareness were significant predictors of intentions to receive the HZ vaccination among residents of Jeddah, Saudi Arabia. Higher education and increased awareness were associated with a higher likelihood of HZ vaccination. Overall, this study underscores the importance of educational interventions in enhancing vaccine uptake among older adults in Saudi Arabia. There is a crucial need to raise public awareness of the benefits of the HZ vaccination. Physicians should also emphasize and deliver knowledge about the significance of vaccination in immunosuppressed patients. Furthermore, organizing educational activities can help clear up misconceptions about vaccine side effects. Overall, collaboration among doctors, healthcare staff, PHC centers, and health field volunteers plays a crucial role in raising awareness about the importance of HZ vaccination.

## Appendices

Data collection form utilized in the study

**Table 7:** Data collection form utilized in the study HZ: herpes zoster Source: This questionnaire was sourced from [13], with additional items added for relevance. Permission to use this questionnaire was obtained from the original publisher

Questionnaire	
First section: demographic data	
Age (years)	1: 10-19 2: 20-29 3: 30-39 4: 40-49 5: 50-59 6: 60-69 7: 70-79 8: ≥80
Gender	1: Male 2: Female
Nationality	1: Saudi 2: Non-Saudi
Educational level	1: Elementary or less 2: High school or less 3: University 4: Higher education
Family history of HZ	1: Yes 2: No
Past history of HZ	1: Yes 2: No 3: Unsure/do not know
Do you remember having had chickenpox?	1: Yes 2: No 3: Unsure/do not know
Have you received the seasonal influenza vaccine in the past year?	1: Yes 2: No
Personal medical history (multiple answers)	1: Hypertension 2: Diabetes mellitus 3: Cancer 4: Gastrointestinal diseases 5: Renal diseases 6: Coronary artery disease 7: Hyperlipidemia
Second section: Awareness of HZ (Shingles) and its vaccine	
Have you ever heard about HZ (Shingles)?	1: Yes 2: No
Do you know anyone with HZ (Shingles)?	1: Yes 2: No
Have you heard of the HZ vaccine, the vaccine for the prevention of shingles?	1: Yes 2: No
Did you receive the HZ vaccine?	1: Yes 2: No
If you didn’t receive the HZ vaccine, are you willing to receive it in the future?	1: Yes 2: No
Do you know that HZ vaccines are recommended for people 50 years and older?	1: Yes 2: No
Have you ever received HZ vaccine recommendations from a healthcare provider?	1: Yes 2: No
Third section: Knowledge of herpes zoster (HZ) (Shingles)	
If an individual had chickenpox, he/she will be at risk of HZ	1: True 2: False 3: Do not know
Immunocompromised individuals are at a higher risk of HZ	1: True 2: False 3: Do not know
Young people will not have HZ	1: True 2: False 3: Do not know
Individuals who have contact with HZ patients will acquire HZ	1: True 2: False 3: Do not know
There are no drugs available for treating HZ	1: True 2: False 3: Do not know
Do you know any symptoms of HZ (more than one can be selected except the last choice)	1: Rash 2: Blisters 3: Neuropathic pain 4: Blindness 5: Hearing loss 6: Death 7: Do not know
Fourth section: Knowledge of herpes zoster (HZ) vaccination	
The HZ vaccine can reduce the incidence of disease by >50%	1: True 2: False 3: Do not know
The HZ vaccine can treat active HZ	1: True 2: False 3: Do not know
Which age group (in years) is approved for vaccination against HZ?	1: No age limit 2: 13-26 3: ≥18 4: ≥50 5: Do not know
